# The Evolving Role of Somatostatin Receptor PET/CT in Medullary Thyroid Carcinoma: An Updated Systematic Review and Meta-Analysis

**DOI:** 10.3390/cancers18132096

**Published:** 2026-06-28

**Authors:** Slavko Tasevski, Alessio Imperiale, Giorgio Treglia, Domenico Albano

**Affiliations:** 1 Nuclear Medicine, University Institute for Positron Emission Tomography, 1000 Skopje, North Macedonia; slavko.tasevski@yahoo.com; 2 Nuclear Medicine, University Hospitals of Strasbourg, University of Strasbourg, 67000 Strasbourg, France; alessio.imperiale@chru-strasbourg.fr; 3 Faculty of Biomedical Sciences, Università della Svizzera Italiana, 6900 Lugano, Switzerland; giorgio.treglia@eoc.ch; 4 Clinic for Nuclear Medicine, Imaging Institute of Southern Switzerland, Ente Ospedaliero Cantonale, 6500 Bellinzona, Switzerland; 5 Faculty of Biology and Medicine, Lausanne University Hospital, 1015 Lausanne, Switzerland; 6 Department of Nuclear Medicine, Università degli Studi di Brescia, 25125 Brescia, Italy; 7 Nuclear Medicine Department, ASST Spedali Civili of Brescia, 25123 Brescia, Italy

**Keywords:** PET/CT, SSTR, medullary thyroid carcinoma, MTC, DOTA

## Abstract

**Background/Objectives**: Medullary thyroid carcinoma (MTC) is a neuroendocrine tumor that often expresses somatostatin receptors (SSTRs). While various PET radiopharmaceuticals were used, there is no universal consensus on the optimal imaging modality for whole-body assessment of MTC. This study aims to evaluate the detection rate (DR) and clinical management impact of SSTR PET/CT imaging in patients with MTC. **Methods**: A systematic search was conducted across PubMed/MEDLINE, Scopus, and Embase. A total of 14 studies (comprising 350 patients) were eligible for quantitative meta-analysis. Pooled DRs were calculated using a random-effects model, and methodological quality was assessed via the QUADAS-2 tool. **Results**: Our analysis revealed an overall DR of 75.1% (95% CI: 67.6–82.6%) for recurrent or metastatic MTC, showing moderate significant heterogeneity (I^2^ = 65.41%). Clinical impact of SSTR PET/CT was demonstrated in 16.6–100% of cases, primarily by identifying candidates for Peptide Receptor Radionuclide Therapy. Only a few studied investigated the relationship between serum calcitonin levels and the detection rate of SSTR PET/CT, finding a significant correlation. **Conclusions**: The DR of SSTR PET/CT in recurrent/metastatic MTC was high. SSTR PET/CT may have a positive impact on clinical management in a significant number of cases.

## Simple Summary

This systematic review and meta-analysis aimed to assess the detection rate and clinical impact of SSTR PET/CT in MTC. The updated meta-analysis revealed a pooled detection rate (DR) of 75.1% (95% CI: 67.6–82.6%) for recurrent or metastatic MTC. SSTR PET/CT influenced patient management in 16.6% to 100% of cases across five studies, primarily by identifying candidates for Peptide Receptor Radionuclide Therapy. Only a few studied investigated the relationship between serum calcitonin levels and the detection rate of SSTR PET/CT, finding a significant correlation.

## 1. Introduction

Medullary thyroid cancer (MTC) is a rare type of thyroid cancer which accounts for 1–8% of all thyroid cancers. It is considered a neuroendocrine tumor arising from parafollicular C cells [1]. MTC produces calcitonin and carcinoembryonic antigen (CEA), which serve as key biological markers in MTC. Most MTC cases are sporadic and 25% of those are hereditary, either familial or in association with multiple endocrine neoplasia type 2, syndrome secondary to mutations in the RET proto-oncogene [2]. MTC has worse prognosis than the differentiated thyroid cancers, due to its aggressive behavior. Up to 50–70% of patients present with cervical lymph node metastases at diagnosis, typically manifesting as a painless neck mass or swelling [3,4]. Increased levels of calcitonin due to tumor presence may also indicate metastatic spread to organs such as in lung, bone, or liver. Therefore, early detection of the primary disease and dissemination is important for appropriate management [5,6]. Ultrasound is the first-line imaging modality for evaluation of thyroid changes that may need further investigation with fine needle aspiration biopsy. Computed tomography and magnetic resonance imaging (MRI) can be used for evaluation of regional or distant metastases. Nuclear medicine techniques enable the early detection of molecular alterations associated with the presence of MTC, often preceding the structural/morphological changes that become apparent later on conventional imaging. Several conventional nuclear medicine imaging techniques have been explored for the detection of locoregional and distant metastases in MTC, although their clinical utility has historically been limited by suboptimal sensitivity [7]. Owing to the superior spatial resolution of positron emission tomography (PET) compared with single-photon emission computed tomography (SPECT), several PET radiopharmaceuticals targeting distinct metabolic pathways and receptor systems have been developed for MTC imaging. Among them, [18F]fluorodihydroxyphenylalanine ([18F]DOPA) and [18F]fluorodeoxyglucose ([18F]FDG) currently represent the most widely adopted PET tracers in clinical practice, particularly for restaging in patients with biochemical recurrence and rising serum calcitonin and/or CEA levels after initial treatment [8,9]. Nevertheless, no single PET tracer provides optimal disease characterization across all clinical scenarios in MTC, reflecting the marked biological disease heterogeneity [8,9].

Given the neuroendocrine origin of MTC, somatostatin receptor (SSTR)-targeted imaging using [68Ga]Ga-labeled DOTA-peptides has also been investigated. Although most MTCs express SSTRs, particularly SSTR2A, receptor density is heterogeneous and generally lower than in well-differentiated neuroendocrine tumors, limiting the routine clinical use of SSTR PET/CT [10]. Consequently, SSTR PET/CT is not currently considered a first-line imaging modality in MTC and is generally reserved for selected clinical scenarios, particularly in patients with negative or equivocal conventional PET imaging, discordant biochemical–imaging findings, or advanced metastatic disease. Importantly, the main added value of SSTR PET/CT in MTC may lie less in its diagnostic superiority than in its theranostic implications, as it enables non-invasive whole-body assessment of SSTR expression and may help identify selected patients who could benefit from peptide receptor radionuclide therapy (PRRT) in advanced metastatic disease [11].

In this updated systematic review, we evaluate the detection rate of somatostatin receptor (SSTR) PET radiotracers in MTC and the clinical impact.

## 2. Materials and Methods

### 2.1. Protocol

We performed this systematic review and meta-analysis of diagnostic accuracy following a rigorous, predefined protocol [12]. This study adheres to the PRISMA guidelines [13]; the finalized checklist is available in Supplementary Materials. While we strictly followed our internal protocol, it was not registered in an external database, as registration remains optional under Item 24 of the PRISMA guidelines [13]. Our research team consists of several experts in SSTR PET/CT and MTC, as well as researchers with methodological expertise in conducting systematic reviews and meta-analyses. To reduce the risk of selection bias, two reviewers (S.T. and D.A.) independently carried out the literature search, study selection, data extraction, and quality appraisal. Any conflicts were resolved through collaborative group consensus. The central review question was formulated using the PICO framework as follows: “What is the diagnostic role (outcome) of SSTR PET/CT (intervention) in MTC (population), when compared or not compared to other imaging methods (comparison)?”

### 2.2. Literature Search and Study Selection

A comprehensive search was performed across PubMed/MEDLINE, Scopus, and Embase to identify relevant studies investigating the role of SSTR PET/CT in MTC patients. The search strategy employed a combination of keywords and Boolean operators, including the following: (1) “positron emission tomography” OR “PET”; (2) “DOTA” OR “SSTR”; AND (3) “medullary” OR “MTC”. No initial date restriction was applied, and the search was updated through 1 April 2026. Additionally, the reference lists of all identified articles were manually screened to capture any further relevant publications.

### 2.3. Data Extraction and Collection

For every included article, data were collected concerning the basic study features (first author name, year of publication, country, funding source, and study design), technical variables (PET scanner used, type of radiopharmaceutical, mean tracer dose injected, uptake time, and kind of analysis), the main clinical patient characteristics (number of patients, age, gender, and calcitonin level), and the main purpose. The main data of the articles included in this review are represented in tables and in Section 3.

### 2.4. Methodological Quality Assessment

The methodological quality of the included studies was independently assessed using the Quality Assessment of Diagnostic Accuracy Studies (QUADAS-2) tool [14]. This validated instrument was employed to evaluate the risk of bias across four primary domains: patient selection, index test, reference standard, and flow and timing. Furthermore, applicability concerns were evaluated for the patient selection, index test, and reference standard domains to determine the clinical relevance of the findings.

### 2.5. Statistical Analysis

For the per-patient analysis, individual study data were synthesized to determine the detection rate (DR) of MTC by SSTR PET/CT. We applied a random-effects model for statistical pooling, reporting the results with 95% confidence intervals (CI) via forest plots. Inter-study heterogeneity was quantified using the I^2^ index, with values exceeding 50% signifying significant heterogeneity. All calculations were executed using OpenMeta [Analyst] software (v. 0.1503, Agency for Healthcare Research and Quality, Rockville, MD, USA).

## 3. Results

### 3.1. Study Selection and Quality Report

Initial database queries identified 210 records after removing duplicates. Following an initial screening of titles and abstracts, 99 records were excluded as they were not related to the field of interest, 10 were excluded as reviews/editorials, 78 were excluded because they were case reports or small case series (less than 5 cases), and 3 were excluded as preclinical studies. Ultimately, 20 studies met the inclusion criteria for the systematic review [15,16,17,18,19,20,21,22,23,24,25,26,27,28,29,30,31,32,33,34] with 14 studies considered eligible for the meta-analysis [16,17,18,19,20,21,22,23,24,25,27,28,31,33]. The other six studies were excluded from the meta-analysis due to the lack of data concerning the detection rate of PET/CT [26,30,34] or a different indication of the scan [15,29,32].

Manual reference tracking yielded no further eligible papers, and all selection decisions reached full consensus among the two reviewers selected (S.T and D.A.).

**Figure 1:**
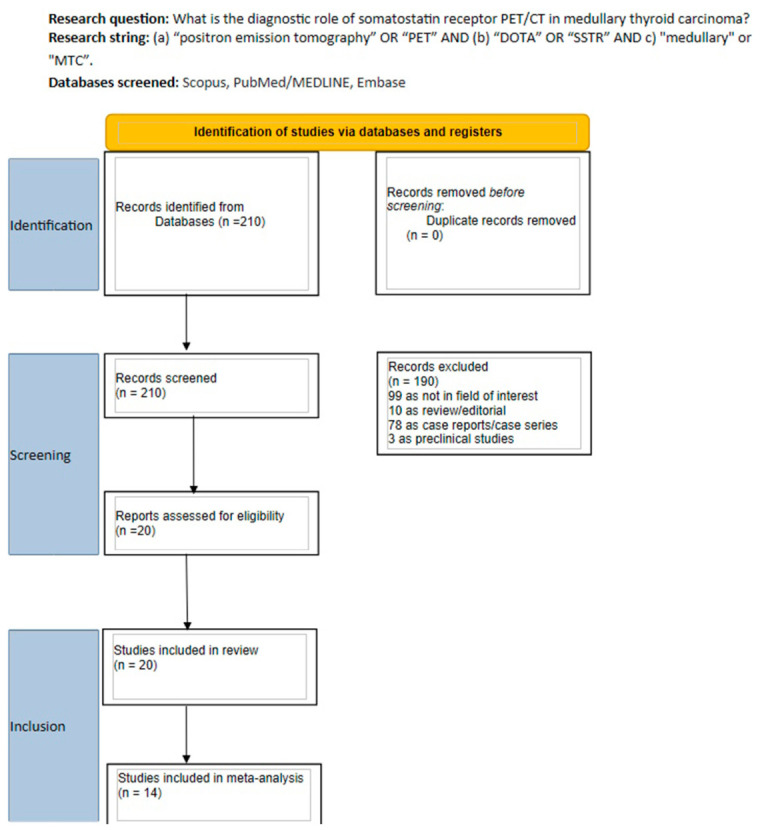
Flow-chart of studies included in our analysis.

**Figure 2:**
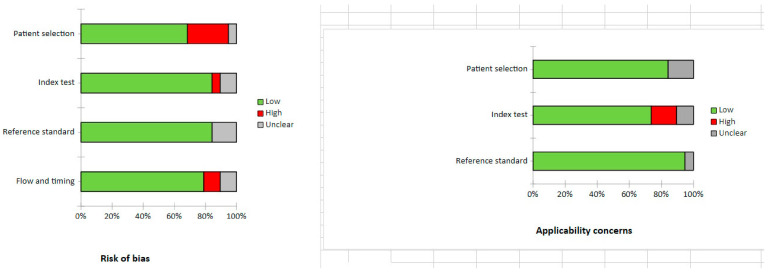
Representation of the QUADAS score of the records included.

The study selection process is detailed in Figure 1 and the results of the quality assessment using the QUADAS-2 tool are shown in Figure 2.

### 3.2. Characteristics of Included Studies and Patients

The main features of the 20 included studies in the systematic review are summarized in Table 1 and Table 2 [15,16,17,18,19,20,21,22,23,24,25,26,27,28,29,30,31,32,33,34]. The selected articles were published between 2010 and 2025, especially in Turkey (*n* = 5), followed by UK (*n* = 3), Germany (*n* = 3), Brazil (*n* = 2), and India (*n* = 2). Funding sources were reported only in three studies. The majority of these studies were retrospective (74%). Participant ages ranged from a median/mean of 42.9–63 years, usually showing a female predominance. The mean/median calcitonin level was very heterogeneous among studies ranging from 133 to 6671.6 pg/mL. Details of the included studies and patient characteristics are summarized in Table 1.

### 3.3. Characteristics of SSTR PET/CT Imaging

In the studies included, different radiopharmaceuticals were used. The most common was [68Ga]DOTA-TATE (*n* = 12), followed by [68Ga]DOTA-NOC (*n* = 3). In four cases, several radiopharmaceuticals were used [18,23,28,29]. In one research [34], [18F]SiTATE was the radiotracer selected. Methodologically, the average injected radiotracer activity varied considerably. When expressed as absolute activities, the administered activity ranged from 72 to 222 MBq; as a relative value, it ranged from 1.5 to 3 MBq/kg. Consistently across all investigations, the time between injection and scan was approximately 60 min (ranging from 45 to 90 min). Technical details of the included studies are described in Table 2. The PET/CT images were analyzed visually in all studies and with the help of semiquantitative parameters in 12 articles [16,17,21,26,27,28,29,30,31,32,33,34]. The most frequent semiquantitative variable used was SUVmax.

### 3.4. Risk of Bias and Applicability

The overall assessment of the risk of bias and concerns about the applicability of the included papers according to QUADAS-2 are provided in Figure 2. Regarding the “Patient Selection” domain, a significant proportion of studies were flagged with a high risk of bias. This is primarily attributable to their retrospective designs and the consecutive inclusion of highly selective, heterogeneous cohorts (such as mixing biochemical relapse with known advanced metastatic patients). Conversely, the “Index Test” and “Reference Standard” domains showed a low risk of bias or low applicability concerns across more than 80% of the analyzed papers, reflecting standardized PET/CT acquisition protocols and robust histopathological or clinical follow-up tracking.

### 3.5. Main Outcomes of SSTR PET/CT Imaging in MTC

For the quantitative analysis, we focused on articles concerning recurrent and metastatic MTC. The primary outcome assessed in the included studies was the diagnostic performances of SSTR PET/CT in recurrent MTC [16,17,18,19,20,21,25,30,31,33], metastatic MTC [24], or both [23,27,28] (Table 3).

Another field investigated was the potential impact on the management of these patients, meaning both from a diagnostic and therapeutic point of view. This aspect was investigated in 6 studies [21,23,27,30,31]. Management impact ranged from 16.6% to 100%, showing high impact in the evaluation of distant localizations of disease. The most important impact of SSTR PET/CT was to guide specific treatments, such as Peptide Receptor Radionuclide Therapy (PRRT).

Moreover, there was a strong relationship between calcitonin value and detection rate of PET/CT in most studies [16,17,21,24,25,27]. Despite qualitative results, in one article, a significant relationship between semiquantitative PET parameters (total tumor burden) and tumor markers was present [25].

### 3.6. Quantitative Evaluation (Meta-Analysis)

**Figure 3:**
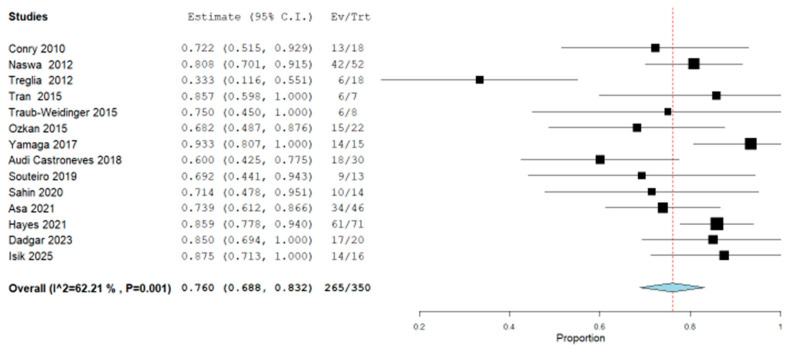
Forest plot of individual studies and pooled detection rate of MTC using 68Ga-SSTR PET/CT, including 95% confidence intervals [16,17,18,19,20,21,22,23,24,25,27,28,31,33]. The size of the squares indicates the weight of each study.

**Figure 4:**
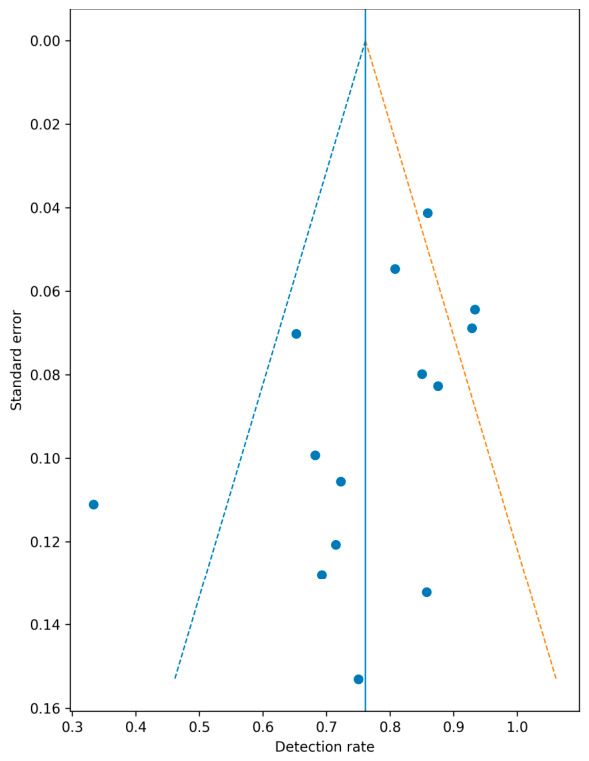
Funnel plot of publication bias.

Fourteen studies including 350 patients with recurrent or metastatic MTC were selected for this bivariate patient-based meta-analysis [16,17,18,19,20,21,22,23,24,25,27,28,31,33]. Results of the meta-analysis are shown in Figure 3. The DR of MTC using SSTR-PET/CT ranged from 33% to 93%, with a pooled outcome measure of 75.1% (95% CI: 67.6–82.6%). There was a moderate heterogeneity among the studies, as indicated by an I^2^ value of 65.41%. To investigate potential publication bias, a funnel plot was generated. Visual inspection of the plot reveals a moderate asymmetry (Figure 4).

## 4. Discussion

The present updated systematic review and meta-analysis provide a comprehensive evaluation of the diagnostic performance and clinical utility of SSTR PET/CT in patients with MTC. Twenty studies were selected for systematic review and 14 were selected for meta-analysis, representing an updated and extended version of the meta-analysis and systematic review previously published [34]. There is no general consensus regarding the use of nuclear medicine imaging in patients with MTC. However, recent recommendations from the European Association of Nuclear Medicine (EANM) support the use of PET/CT imaging techniques in patients with elevated MTC tumor marker levels after surgery, particularly when morphological imaging is negative or inconclusive [9]. Notably, all included studies involved patients with increased median calcitonin levels, which provides a relevant clinical basis for assessing the diagnostic performance of somatostatin receptor PET/CT in patients with a high suspicion of recurrent disease. In our meta-analysis, we focused on the pooled DR, as a meta-analysis of sensitivity and specificity was not feasible due to limited reporting and methodological heterogeneity across studies. In particular, insufficient data for reconstructing contingency tables and variability in patient-, lesion-, and region-based analyses precluded a reliable bivariate diagnostic accuracy analysis. The pooled DR was 75% with acceptable consistency across studies (I^2^ = 65.41%). This value is higher than that reported by Treglia et al., which was 63.5% [34], likely reflecting the inclusion of more recent studies and advancements in imaging technology. In Treglia et al.’s study, only nine studied were included for a total of 152 patients. Despite this, approximately 21.5% of patients remained undetected, which may be explained by heterogeneous SSTR2 expression, low tumor burden, or tumor dedifferentiation. Compared with other functional imaging modalities, SSTR PET/CT demonstrated a good detection rate despite the lack of direct comparisons with other radiotracers. A meta-analysis focus on [18F]-DOPA PET/CT showed a DR of 66% [35]. These modalities should therefore be considered complementary, with SSTR PET/CT potentially performing better in tumors with preserved receptor expression [9,36].

A significant contributor to the moderate inter-study heterogeneity (I^2^ = 65.41%) is the difference in radiotracer selection and patient disease stages. The included studies utilized several radiophgarmaceuticals, some 68Ga-labeled peptides (DOTATE, DOTANOC, DOTATOC) which may exhibit slightly different binding affinities across somatostatin receptor subtypes (SSTR2, SSTR3, and SSTR5). Furthermore, the recent introduction of the cyclotron-produced18F-SiTATE by Kunte et al. [33] introduces an entirely different radiopharmacokinetics profile.

To contextualize the findings of this meta-analysis, it is essential to define where SSTR PET/CT is localized in the diagnostic chart of MTC. This is not considered a first-line diagnostic modality for MTC. DOPA PET/CT remains widely recognized as the most sensitive functional imaging method for localizing disease in the setting of biochemical recurrence, while [18F]FDG PET/CT plays a critical and complementary prognostic role. Patients with high FDG uptake typically are correlated with biochemical progression, disease dedifferentiation, and aggressive tumor biology, helping to identify patients at a high risk of rapid progression. In this scenario, SSTR PET/CT might have the crucial role of identifying patients who are candidates for PRRT.

Although most of the included studies suggested a correlation between calcitonin levels and the detection rate of PET/CT, only a minority formally assessed this relationship using statistical analysis. In our dataset, only 6 out of 15 studies performed a statistical evaluation of this association, which limits the strength of this conclusion. Therefore, while this relationship is biologically plausible and consistently observed, its quantitative strength remains insufficiently validated across studies. However, the demonstration of a positive correlation between elevated serum calcitonin levels and higher SSTR PET/CT detection rates is crucial. Biologically, serum calcitonin directly mirrors total neuroendocrine tumor burden and secretory activity in MTC [37]. In patients with very low or slowly doubling calcitonin levels, recurrent disease may consist of sub-centimeter lymph nodes or low-density tumor aggregates that can be difficult to detect by PET/CT imaging. Instead, markedly elevated calcitonin levels signify a higher pre-test probability of macro-metastatic spread (e.g., to the liver, bones, or mediastinum), rendering these lesions highly detectable. Clinically, this finding may suggest that SSTR PET/CT yields its highest diagnostic performance and cost-effectiveness when reserved for patients displaying significant biochemical recurrence or rapid calcitonin doubling times, rather than minimal, stable baseline elevations.

The clinical impact of SSTR PET/CT on patient management was reported in five studies with a very heterogeneous interval ranging from 18.2% to 100%. This wide range likely reflects differences in study design, sample size, patient selection (some mixed cohorts), and definitions of “management impact” that is strongly affected by individual point of view. The same definition of “management impact” is very heterogenous among studies; we conventionally decided to consider it as a documented change in patient management that led to a modification in the diagnostic and/or treatment plan (e.g., switching from localized surgical intent to systemic therapy, identifying previously occult distant metastases, or avoiding unnecessary invasive procedures). Nevertheless, SSTR PET/CT may influence clinical decision-making, particularly by identifying candidates for PRRT.

In our systematic review, a subset of studies specifically evaluated how SSTR PET/CT directly guided therapeutic strategy or PRRT eligibility. For instance, Hayes et al. [27] and Dadgar et al. [30] evaluated the direct feasibility and selection of patients for 177Lu-DOTATATE therapy based on tracer avidity. Furthermore, publications by Ozkan et al. [20], Souteiro et al. [23], and Asa et al. [26] demonstrated that positive SSTR findings directly shifted management toward PRRT or surgical intervention in patients with advanced disease. However, because prospective, randomized controlled data with survival endpoints remain limited, SSTR PET/CT should not be generalized as a universal therapeutic guide, but rather as a highly specialized tool for selecting candidates for PRRT within clinical trials or specialized salvage protocols.

This theranostic role represents a major advantage over purely diagnostic imaging modalities, positioning SSTR PET/CT as a key tool in personalized medicine for MTC [11]. However, the current experiences about PRRT with radiolabeled SSA in MTC are limited. Consequently, at this time, PRRT should only be considered in selected patients in the context of clinical trials, because more solid data from randomized controlled studies with survival endpoints are needed [38].

Another potential role of SSTR PET/CT in MTC that has not well investigated is the usefulness in evaluating serial SSTR PET/CT for the detection of dynamic changes in SSTR expression and consequently in the guiding of treatment response (such as PRRT or systemic therapies).

Recently, a new SSTR radiotracer [18F]SiTATE was introduced by Kunte et al. [33]. Unlike [68Ga]-labeled compounds, which rely on generator-based production, these [18F]-labeled tracers are cyclotron-produced and therefore may offer logistical and economic advantages, particularly in the context of patient selection for PRRT. However, more solid data are necessary to really understand the potentiality of this tracer.

This study has several limitations. Most included studies were retrospective with relatively small sample sizes, reflecting the rarity of MTC and potentially introducing selection bias. A formal meta-analysis of sensitivity and specificity was not feasible due to limited and heterogeneous reporting, including variability between patient-based, lesion-based, and region-based analyses; therefore, detection rate was used as the primary outcome, which may not fully reflect diagnostic accuracy. Significant heterogeneity was present in patient characteristics, calcitonin levels, disease stage, and imaging protocols. Although a correlation between calcitonin and detection rate was consistently observed, it was not uniformly validated statistically. Furthermore, variability in radiotracers and image analysis methods limits comparability across studies. Data on clinical impact were limited and inconsistently defined, and cost-effectiveness could not be assessed.

A major inherent limitation of this meta-analysis and of the current body of the literature concerning SSTR PET/CT in MTC is the substantial clinical heterogeneity within the studied cohorts. The pooled DR derived by this meta-analysis reflects a mixed population of patients with recurrent disease and metastatic MTC. These clinical scenarios may present different pre-test probabilities and tumor burdens, which can affect functional imaging performances.

## 5. Conclusions

Overall, SSTR PET/CT represents a valuable imaging modality in MTC patients with recurrent or metastatic disease, particularly in those with higher tumor burden and preserved receptor expression, while also playing a key role in guiding theranostic approaches.

## Figures and Tables

**Table 1:** Main features of the studies selected.

First Author	Year	Country	Funding Source	Study Design	N° Patients	Age Mean (Range)	Gender M (%)	Calcitonin, Median/Mean (Range), pg/mL	Purpose/s
Palyga I [15]	2010	Poland	None reported	R	8	55.6 (41–72)	4 (50%)	265 (53–720)	Staging in patients with negative conventional imaging (ultrasound, CT, MRI, FDG PET)
Conry BG [16]	2010	UK	None reported	R	18	54 (34–75)	13 (72%)	134.9 (1.5–550)	Comparison with FDG PET/CT in recurrent MTC
Naswa N [17]	2012	India	None reported	P	52	44.7 (18–73)	38 (73%)	4017.3 (50–30,000)	Comparison with FDG PET/CT in recurrent MTC
Treglia G [18]	2012	Italy	Non reported	R	18	53 (24–86)	6 (33%)	527 (66.7–14,186)	Comparison with FDG PET/CT and DOPA PET/CT in recurrent MTC
Tran [19]	2015	UK	None reported	R	7	45 (31–66)	3 (42%)	8320 (672–37,180)	Detection rate in recurrent MTC
Traub-Weidinger [20]	2015	Austria	None reported	R	31 *	63 (24–85)	20 (64%)	na	Detection rate in recurrent MTC
Ozkan ZG [21]	2015	Turkey	None reported	R	22	42.9(20–69)	11 (50%)	871.5 (99.4–7370)	Comparison with FDG PET/CT and DMSA scintigraphy in recurrent MTC
Yamaga [22]	2017	Brazil	None reported	P	15	43.6 (20–68)	6 (40%)	10.990 (417–100,000)	Comparison with 111ln-Octreotide scintigraphy and conventional imaging in recurrent MTC
Audi Castroneves L [23]	2018	Brazil	None reported	P	30	48 (19–78)	13 (43%)	133 (12–1162) in staging group 8323 (564–101,083) in metastatic group	Diagnostic performances and comparison with CT, MRI, and bone scan in metastases detection
Souteiro P [24]	2019	Portugal	None reported	R	13	50.9 (39–78)	5 (38%)	828 (20.4–231,696)	Comparison with FDG PET/CT in metastatic MTC
Sahin E [25]	2020	Turkey	None reported	R	14	54.4 (27–81)	6 (43%)	1201.2 (24.3–7292)	Comparison with FDG PET/CT in recurrent MTC and correlation with tumor markers (calcitonin and CEA)
Arici S [26]	2021	Turkey	None reported	R	28	55 (35–82)	9 (32%)	6671.6 (2–102,148)	Correlation between tumor burden and tumor markers (calcitonin and CEA)
Asa S [27]	2021	Turkey	yes	P	46	53 (27–82)	21 (46%)	2032 (62.4–14,000)	Comparison with 18F-DOPA PET/CT in recurrent and metastatic MTC
Hayes [28]	2021	UK	yes	R	71	44 (16–84)	37 (52%)	3477 (6.8–174,000)	Detection rate in metastatic and recurrent MTC and eligibility for PRRT
Serfling SE [29]	2022	Germany	None reported	R	23	51.9 (32–81)	12 (52%)	1400 (26–42,700)	Staging and therapeutic decision
Ballal S [30]	2023	India	None reported	R	27	42.4 (14–66)	21 (78%)	666.5 (389–1145)	Comparison with FAPI PET/CT in follow-up
Dadgar H [31]	2023	Iran	None reported	R	20	48.5 (27–71)	10 (50%)	256 (14–2000)	Restaging in suspected relapse
Gild ML [32]	2024	Australia	None reported	R	37	na	na	na	Prognostication
Isik EG [33]	2025	Turkey	None reported	R	16	50 (18–76)	8 (50%)	6234 (245–96,880)	Comparison with FAPI PET/CT in recurrent MTC
Kunte SC [34]	2025	Germany	yes	P	21 **	62.1 (40–73)	4 (40%)	na	Feasibility

R—retrospective; P—prospective; na—not available; CT—computed tomography; MTC—medullary thyroid carcinoma; PRRT—peptide receptor radionuclide therapy. *, 8 MTC. **, 10 MTC.

**Table 2:** Technical characteristics of studies included.

First Author	Device	Radiopharmaceutical	Mean Radiotracer Injected Dose, MBq	Mean Uptake Time (Min)	Image Analysis	Semiquantitative Variables
Palyga I [15]	PET/CT	[68Ga]DOTA-TATE	120–185	60	Visual	
Conry BG [16]	PET/CT	[68Ga]DOTA-TATE	90–220	45–60	Visual and semiquantitative	SUVmax
Naswa N [17]	PET/CT	[68Ga]DOTA-NOC	148–222	45–60	Visual and semiquantitative	SUVmax
Treglia G [18]	PET/CT	[68Ga]DOTA-NOC *n* = 14	1.5–2/kg	60	Visual	
		[68Ga]DOTA-TOC *n* = 4	2.5/kg			
Tran [19]	PET/CT	[68Ga]DOTA-TATE	110–148	45–60	Visual	
Traub-Weidinger [20]	PET/CT	[68Ga]DOTA-TATE	185	60	Visual	
Ozkan ZG [21]	PET/CT	[68Ga]DOTA-TATE	185	nr	Visual and semiquantitative	SUVmax
Yamaga [22]	PET/CT	[68Ga]DOTA-TATE	72	45	Visual	
Audi Castroneves L [23]	PET	[68Ga]DOTA-LAN & [68Ga]DOTA-TOC	95–150 100–150	90	Visual	
Souteiro P [24]	PET/CT	[68Ga]DOTA-NOC	122	76	Visual	
Sahin E [25]	PET/CT	[68Ga]DOTA-TATE	2–3/kg	45	Visual	
Arici S [26]	PET/CT	[68Ga]DOTA-TATE	2/kg	60	Visual and semiquantitative	MTV, TLV
Asa S [27]	PET/CT	[68Ga]DOTA-TATE	176	45–60	Visual and semiquantitative	sumSUVmax
Hayes [28]	PET/CT	[68Ga]DOTA-TATE & [68Ga]DOTA-TOC & [68Ga]DOTA-NOC	nr	nr	Visual and semiquantitative	SUVmax
Serfling SE [29]	PET/CT	[68Ga]DOTA-TATE & [68Ga]DOTA-TOC	120	60	Visual and semiquantitative	SUVpeak, TV
Ballal S [30]	PET/CT	[68Ga]DOTA-NOC	185	60	Visual and semiquantitative	SUVpeak, TBR
Dadgar H [31]	PET/CT	[68Ga]DOTA-TATE	148–185	60	Visual and semiquantitative	SUVmax
Gild ML [32]	PET/CT	[68Ga]DOTA-TATE	120–180	50	Visual and semiquantitative	SUVmax, SUVmean, MTV and TLA
Isik EG [33]	PET/CT	[68Ga]DOTA-TATE	185	60	Visual and semiquantitative	SUVmax
Kunte SC [34]	PET/CT	[18F]SiTATE	215.5	90	Visual and semiquantitative	SUVmax, SUVmean, TTV, wb-SUV

nr—not reported; SUV—standardized uptake value; MTV—metabolic tumor volume.

**Table 3:** Diagnostic performances of SSTR PET/CT in MTC on a patient-based analysis.

First Author	PET Positive/Total PET (%)	Mean SUVmax	Management Impact *n* (%)	Kind of Impact	Correlation with Calcitonin Levels
Conry BG [16]	13/18 (72%)	na	na	na	yes
Naswa N [17]	42/52 (81%)	Local recurrence: 3 Cervical metastasis: 3.4 Mediastinal node metastasis: 6.2 Liver metastasis: 6 Skeletal metastasis: 12.9	na	na	>500 pg/mL (lack significance analysis)
Treglia G [18]	6/18 (33%)	na	3 (16.6%)	Guide to biopsy	yes
Tran [19]	6/7 (86%)	na	na	Confirmation or ruling out recurrence	na
Traub-Weidinger [20]	6/8 (75%)	na	na	na	na
Ozkan ZG [21]	15/22 (68%)	na	18.20%	Guide to surgery and PRRT	yes
Yamaga [22]	14/15 (93%)	na	na	na	na
Audi Castroneves L [23]	5/16 (31%) in a biochemical recurrence group; 13/14 (93%) in a metastatic disease group	6.4 (median) in a metastatic disease group	6.25% in a biochemical recurrence group; 35.7% in a metastatic disease group	Initiation of treatments such as bone antiresorptives, radiation therapy, or a change of radiological workup during follow-up.	na
Souteiro P [24]	9/13 (69%)	na	na	Guide to surgery or PRRT	yes
Sahin E [25]	10/14 (71%)	na	na	na	yes (with total tumor burden)
Asa S [27]	30/46 (65%)	31.1 *	68.40%	Guide to surgery, PRRT, RFA or combined treatment.	yes
Hayes [28]	61/71 (86%)	na	na	na	na
Ballal S [30]	na	na	94.4% for primary tumours 95% for lymph nodes 100% for brain metastases 68.9% for lung nodules 46.4% for liver metastases 76.5% for bone metastases 0% for pleural metastases	na	na
Dadgar H [31]	17/20 (85%)	8.77	20%	Guide to PRRT	na
Isik EG [33]	14/16 (87.5%)	9.7	na	na	na

na—not available; * sumSUVmax; PRRT—Peptide Receptor Radionuclide Therapy; RFA—radiofrequent ablation.

## Data Availability

The datasets used and analyzed during the current study are available from the corresponding author on reasonable request.

## Supplementary Materials

The following supporting information can be downloaded at: https://www.mdpi.com/article/10.3390/cancers18132096/s1, PRISMA 2020 Checklist.

## Abbreviations

The following abbreviations are used in this manuscript:

MTC	medullary thyroid carcinoma
SSTR	somatostatin receptors
DR	detection rate
PET/CT	positron emission tomography/computed tomography
CEA	carcinoembryonic antigen
PRRT	Peptide Receptor Radionuclide Therapy
CI	confidence intervals
SPECT	single-photon emission computed tomography
